# COVID-19, Hypertriglyceridemia, and Acute Pancreatitis: A Case Report and Clinical Considerations

**DOI:** 10.7759/cureus.35431

**Published:** 2023-02-24

**Authors:** Abdelwahab Jalal Eldin, Anthony Lyonga, Biola Ohiokpehai, Muhammad Rizwan, Abdullahi Musa

**Affiliations:** 1 Internal Medicine, Howard University Hospital, Washington, DC, USA; 2 Pulmonary Medicine, Howard University Hospital, Washington, DC, USA; 3 Gastroenterology, Howard University Hospital, Washington, DC, USA

**Keywords:** covid-19, hypertriglyceridemic pancreatitis, acute pancreatitis, covid-19 and pancreatitis, pancreatitis in infections

## Abstract

Acute pancreatitis (AP) is a serious condition that can result in numerous negative outcomes including death. The underlying causes of AP are varied, with both COVID-19 and hypertriglyceridemia being documented in the medical literature. Here, we present the case of a young man with a pre-existing diagnosis of prediabetes and class 1 obesity who developed severe hypertriglyceridemia, AP, and mild diabetic ketoacidosis while concurrently infected with COVID-19. It is crucial for healthcare providers to be vigilant in recognizing the potential complications associated with COVID-19, regardless of whether the patient has received a vaccination.

## Introduction

Viral pancreatitis is a rare but well-known complication of many viral infections, including hepatitis B, mumps, Coxsackievirus, and others [1]. Recent reports have suggested a potential association between severe acute respiratory syndrome coronavirus 2 (SARS-CoV-2) and acute pancreatitis (AP) [2].

The exact mechanisms involved in the development of pancreatitis in individuals with coronavirus disease 2019 (COVID-19) are yet to be fully understood and may include direct pancreatic injury through the angiotensin-converting enzyme (ACE 2) receptor, mediator release, diffuse severe endotheliitis, systemic inflammatory response, and drug-related pancreatic injury [3].

Patients with SARS-CoV-2 infection have also been reported to experience hyperglycemia, which can be a complication of pancreatic injury [4,5]. Additionally, hypertriglyceridemia has been observed in some COVID-19 patients, with most cases attributed to medication use. However, a small number of patients with careful medical follow-up have developed new-onset hypertriglyceridemia during the active or recovery phase of the infection [6]. To our knowledge, this is the first case of severe hypertriglyceridemia and COVID-19 infection in a fully vaccinated individual resulting in AP [7,8].

## Case presentation

A 31-year-old African American male with a history of prediabetes and class 1 obesity presented to the emergency department (ED) complaining of a two-day history of severe, constant, non-radiating right upper quadrant abdominal pain associated with nausea and a decrease in appetite. He also reported polyuria, polydipsia, and bilateral hand numbness. He was vaccinated for COVID-19. He drank liquor on weekends. His last drink was four days prior. Physical examination was significant for upper abdominal tenderness. Lab workup showed elevated lipase, blood glucose, anion gap, and serum creatinine, as shown in Table 1.

**Table 1:** Laboratory investigations. AG: anion gap; BUN: blood urea nitrogen; Cr: creatinine; Ca: calcium, BHB: beta-hydroxybutyrate; ALP: alkaline phosphatase; ALT: alanine transaminase; AST: aspartate transaminase; LDH: lactate dehydrogenase; CRP: C-reactive protein; INR: international normalized ratio; HCT: hematocrit

Investigation	Result on admission	At 48 hours	Reference
Serum lipase	280		10–60 IU/L
Bicarbonate	19		22–32 mEq/L
Serum AG	15.3		3.5–11 mEq/L
BUN	13	8	7–25 mg/dL
Serum Cr	1.47		0.6–1.2 mg/dL
Corrected Ca	9.1	9.4	8.5–10.3 mg/dL
Serum glucose	443		70–100 mg/dL
BHB	28.1		0–4.5 mg/dL
HbA1c	10.1		<5.7%
Ferritin	463.7		20–400 mEq/L
CRP	10.6		<10 mg/dL
ALP	94		30–130 IU/L
ALT	28		0–55 IU/L
AST	34		0–50 IU/L
Total bilirubin	0.6		0.2–1.2 mg/dL
LDH	242		100–250 IU/L
Ethanol, blood	<10		<10 mg/dL
Triglyceride level	2,516	409	<150 mg/dL
WBC	13.8		3.2–10.6 × 10^9^/L
HCT	45.9	38.3	40.8–51.9%

**Figure 1:**
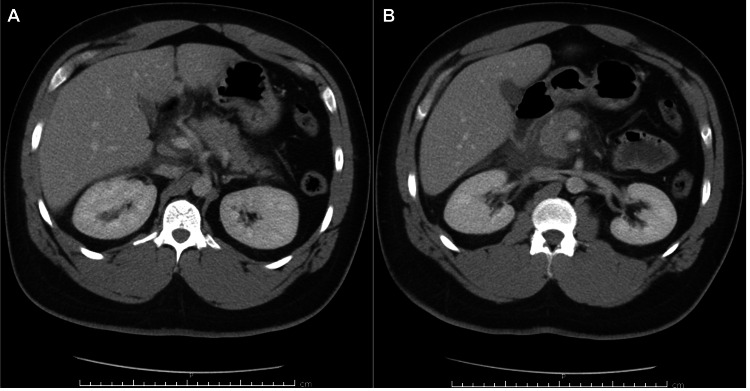
(A) Prominent pancreas with retroperitoneal free fluid. (B) Inflammation/fluid around the head and uncinate process of the pancreas.

Contrast-enhanced CT of the abdomen was consistent with AP (Figure 1), and abdominal ultrasound did not show any gallstones. He had a Ranson score of 1 on admission and at 48 hours, and a modified Marshall score of 0.

The patient was initially managed with aggressive hydration and subcutaneous insulin. His serum anion gap closed within four hours. When his triglycerides were found to be elevated at 2,516 mg/dL, he was placed on an intravenous insulin drip and admitted to the medical care intensive unit (MICU). The endocrine team was consulted for new-onset diabetes and diabetic ketoacidosis (DKA). On admission to the MICU, he started having a fever with a temperature of 102.7°F, tachycardia of 119, and a white blood cell count of 11.2, with 19% band cells. He was then started on broad-spectrum antibiotics. His SARS-CoV-2 polymerase chain reaction test was positive. His blood cultures were negative. He maintained an SpO_2_ of >92% and did not require supplemental oxygen. The patient was successfully bridged to weight-based subcutaneous insulin after his triglyceride (TGL) level was down to less than 500 mg/dL; subsequently, he was started on a fat-restricted diet when he started tolerating food. Throughout the hospital course, the patient maintained good hemodynamics with a blood pressure above 120/80 mmHg.

## Discussion

Multiple studies have reported pancreatic injury in COVID-19 infection [2]. COVID hypertriglyceridemic pancreatitis (HTG-AP) has only been reported in a handful of cases [8]. We report the first case of HTG-AP in a fully vaccinated COVID-19-positive individual. Hypertriglyceridemia can be familial or secondary to uncontrolled diabetes, medication use, or alcohol use. Hypertriglyceridemia has been reported as a complication of COVID-19. Our patient did not have any family history of hyperlipidemia, had undetectable blood ethanol, and was not taking any medication. HTG-AP in DKA is usually seen with severe metabolic acidosis [9]. However, our patient presented with a mildly elevated anion gap. Moreover, DKA potentially causes a moderate or mild elevation in TGL. Our patient presented with severe hypertriglyceridemia (TGL >1,000 mg/dL) [10].

COVID-19 infection often presents with local and systemic manifestations, and many reports and reviews have highlighted the coexistence of COVID-19 and AP. A narrative review conducted on published articles in nearly two years reported a higher incidence of idiopathic AP in patients with COVID-19. However, we still do not have conclusive evidence to list COVID-19 as an independent cause of AP [11]. Torres et al. reported a similar case presentation of hyperglycemia, hypertriglyceridemia, AP, and COVID-19 infection, in which the patient required mechanical ventilation and had a prolonged hospital course [8].

Hypertriglyceridemia is the third most common cause of AP and accounts for about 10% of the cases [12,13]. Young males appear to be the most commonly affected individuals (excluding gestational pancreatitis) [14,15]. The incidence of HTG-AP in a COVID-19-positive individual should not be considered a mere coincidence. Transient inhibition of lipoprotein lipase was observed following a COVID-19 infection. This culminates in elevated TGL. It is then through hydrolysis of excessive triglycerides by pancreatic lipase and excessive formation of free fatty acids that lead to inflammation and capillary and pancreatic injury [6,13].

Treatment for HTG-AP includes decreasing TGL using insulin, plasmapheresis, or a combination of both. The American Society for Apheresis recommends therapeutic plasma exchange for patients with severe HTG-AP [16]. Our patient did not meet the criteria for severe pancreatitis, and as insulin was readily available, he responded within a few hours with a steady reduction in TGL and an improvement in symptoms. He was initially managed with aggressive intravenous hydration as he did not test positive for SARS-CoV-2 until day three. Had the patient had severe COVID-19 infection with acute respiratory distress syndrome it remains a question whether the conventional aggressive intravenous hydration would have been an optimum choice of management.

Higher TGL confers a higher risk of AP and correlates with the severity of the disease and organ failure. Contrary to multiple reports, our patient presented with TGL of >2,500 mg/dL, his hospital course included admission to the MICU, but no complications were observed [14,17-19]. It is unclear whether being vaccinated played a role in the severity of pancreatitis.

Infectious pancreatitis constitutes about 10% of the causes; the vast majority are caused by viral infections including hepatitis B, mumps, Coxsackievirus, cytomegalovirus, and human immunodeficiency virus [1]. There have been some questions about the causality and whether SARS-CoV-2 can cause AP. Owing to the difficulty of undergoing endoscopic ultrasound and sometimes MRI to exclude other causes of pancreatitis while the patient remains positive for SARS-CoV-2 RNA, we still do not have enough evidence to lay the blame solely on SARS-CoV-2 infection [1,2,20]. Nonetheless, the evidence is increasing and should only encourage us to remain vigilant with a high index of suspicion for pancreatitis in COVID-19 patients who present with abdominal pain regardless of vaccination status, as seen in our patient, while also investigating other causes of pancreatitis to avoid inappropriate management [20].

## Conclusions

There is evidence to suggest that COVID-19 may be a contributing factor to the development of hypertriglyceridemia and AP. Further investigation through well-designed longitudinal studies with large sample sizes is required to fully elucidate the mechanisms involved and the impact of vaccination on this association. Clinicians should be aware that abdominal pain unrelieved by typical measures in patients with COVID-19 may indicate AP and prompt further evaluation should be pursued.
